# Patterns and determinants of tobacco purchase behaviors among male cigarette smokers in Vietnam: A latent class analysis

**DOI:** 10.18332/tid/187869

**Published:** 2024-06-04

**Authors:** Thi Ngoc Phuong Nguyen, Monica Hunsberger, Jesper Löve, Tu Anh Duong, Thi Hai Phan, Ngoc Khue Luong, Van Minh Hoang, Nawi Ng

**Affiliations:** 1 Institute of Medicine, Sahlgrenska Academy, University of Gothenburg, Gothenburg, Sweden; 2 Center for Population Health Sciences, Hanoi University of Public Health, Hanoi, Vietnam; 3 Ministry of Health, Hanoi, Vietnam; 4 Department of Epidemiology and Global Health, Faculty of Medicine, Umea University, Umea, Sweden

**Keywords:** cigarette, purchase behavior, policy, latent class analysis

## Abstract

**INTRODUCTION:**

Understanding smokers’ purchasing patterns can aid in customizing tobacco control initiatives aimed at reducing the tobacco smoking prevalence. Therefore, this study identified cigarette purchase behavior among Vietnamese male smokers and associated demographic and consumption factors.

**METHODS:**

We analyzed a secondary dataset of male current tobacco smokers (n=3983) who participated in the Vietnam Global Adult Tobacco Survey in 2015. We applied the latent class analysis (LCA) to identify the classes of purchase behavior among cigarette smokers (n=1241). Multinomial logistic regression was performed to identify demographics (education level, ethnicity, partnership status, and household socioeconomic status) and cigarette consumption variables (smoking years and heavy smoking status) related to purchase behavior classes. The results are reported as an adjusted relative risk ratio (ARRR).

**RESULTS:**

The LCA identified four cigarette purchase behaviors classes: Class 1 (price-insensitive and purchased international brand: 44.4%), Class 2 (price-sensitive and purchased domestic brand: 27.6%), Class 3 (price-sensitive and purchased cigarettes in a street vendor: 18.6%), and Class 4: price-sensitive and purchased loose/carton cigarette: 9.4%). The poorer economic groups were more likely to belong to the three price-sensitive classes. Heavy smokers and those who had smoked for a longer period were more likely to belong to Class 3 (ARRR=2.33; 95% CI: 1.51–3.58 and ARRR=1.02; 95% CI: 1.001–1.05, respectively) and Class 4 (ARRR=2.94; 95% CI: 1.71–5.06 and ARRR=1.05; 95% CI: 1.02–1.08, respectively).

**CONCLUSIONS:**

Varied purchasing behaviors among male cigarette smokers, influenced by divergent price sensitivities and economic backgrounds, underscore the need for comprehensive tobacco control. Future efforts should include targeted policy interventions, behavior modification, and reshaping social norms.

## INTRODUCTION

Tobacco-related diseases are a significant health burden worldwide; over 8 million deaths are attributed to tobacco use annually^1^. With over 80% of global tobacco users living in low- and middle-income countries, these nations face a significant burden of tobacco-induced illnesses and related deaths^1^. Tobacco consumption exacerbates poverty by causing a diversion of household funds from essential needs, such as food and shelter, towards tobacco^1^.

Vietnam is a middle-income country where approximately 22.5% of adults aged ≥15 years consume some form of tobacco^2^. The prevalence is much higher among men (45.3%) than women (1.1%), according to a 2015 survey^2^. The majority of male tobacco users smoke cigarettes (80.0%), followed by traditional waterpipe users (30.0%)^2^. Tobacco-related diseases led to >112000 deaths in Vietnam in 2019, contributed significantly to healthcare costs, and threatened national development goals^3^.

The popularity, availability, and affordability of cigarettes make the battle on tobacco use hard to fight^4^. In Vietnam, the government-owned tobacco companies supplied 80% of the cigarette market (both domestic and legally imported cigarettes), while the remaining 20% was illegal cigarettes obtained through cross-border trading with the neighboring countries^5^. Moreover, the tobacco industry also promotes cigarettes of various brands, flavors, and prices to increase their availability and aims to reach all income levels and flavor profiles in Vietnam. In terms of affordability, the mean expenditure for a pack of 20 manufactured cigarettes was VND 11819 in 2015 (about US$0.48), lower than the estimated cost in 2010 after adjusting for inflation^2^. Further, the World Health Organization (WHO) reported that cigarette prices in Vietnam have become more affordable since 2010^6^.

To combat tobacco use, Vietnam has significantly strengthened tobacco control policies in the last decade, such as the implementation of pictorial health warnings on cigarette packs, raising tobacco taxes, and setting minimum cigarette prices^7,8^. However, the national smoking prevalence remained high and was nearly stagnant between 2010 and 2015 among men^2^. Therefore, this study aimed to identify patterns of tobacco purchase behavior among Vietnamese male cigarette smokers and the associated demographic and consumption factors. Understanding the purchasing patterns can aid in customizing tobacco control initiatives aimed at reducing the prevalence of cigarette smoking in Vietnam in the coming time.

## METHODS

### Study sample and setting

For this secondary analysis we used the dataset of the 2015 Global Adult Tobacco Survey (GATS) cross-sectional survey performed in Vietnam. The GATS is a nationally representative survey that aims to systematically monitor tobacco use and track the effectiveness of tobacco control policies among adults aged ≥15 years^9^. This survey was designed as a two-stage stratified sampling, with enumeration area (EA) as the primary and household as the secondary sampling units. About 10% of households in each selected EA were included, and one individual in each sampled household was randomly chosen for interview using the Kish method^10^. This method randomly selects household survey participants and helps mitigate selection bias, typically arising from improper participant selection procedures.

### Ethics

This study is based on analyzing the open-access survey data with all identifier information removed to ensure anonymity. The Global Adult Tobacco Survey (GATS) Vietnam 2015 was approved by the Ministry of Health of Vietnam. The Vietnam Steering Committee on Smoking and Health of the Ministry of Health was the lead agency to conduct this survey, and the General Statistics Office of Vietnam collected the data. Informed consent was obtained from all study participants before participation in interviews and ethical principles were followed at all stages of the survey. The raw data from GATS Vietnam 2015 were used for this study after securing the required permissions.

### Participants

To be eligible for the study, participants had to be aged ≥15 years and be non-institutionalized residents in the survey areas of Vietnam. Those aged <15 years, non-citizens, visitors, enlisted military personnel, and institutionalized people (including those residing in hospitals, prisons, nursing homes, and other institutions) were excluded. A total of 8996 eligible individuals were selected. Since the prevalence of tobacco smoking among women in Vietnam is low (around 1.0%), we only included male participants in our analysis (n=3983). The study design and methodology have been reported elsewhere^2^.

### Measures

*Tobacco smoking behavior*

Participants were asked to respond to the questions: ‘Do you currently smoke tobacco either daily, less than daily, or not at all?’. The respondents were categorized as current smokers or current non-smokers (ex-smokers or never smokers). Respondents who smoked currently were asked to indicate the types of tobacco they used, which included cigarettes, hand-rolled cigarettes, pipes, cigars, waterpipes, or shisha.

*Purchase behavior*

Of those who smoked cigarettes (n=1518), GATS assessed their most recent purchase behaviors using the following items: the quantity purchased (loose, pack, or carton), the brand purchased (domestic or international), the price of cigarette purchased, and the location of purchase (kiosk, street vendor or other). We categorized cigarette price tiers according to the definitions of the Vietnam Ministry of Finance defined as follows: low (<10000 VND/pack, equivalent to <US$0.4/pack), medium (10000–22500 VND/pack, equivalent to US$0.4–0.9/pack), and high (>22500 VND/pack, equivalent to >US$0.9/pack) based on the range of retail prices of all cigarettes in the market in 2015. The subsequent analyses were focused on cigarette smokers.

*Covariates*

The covariates in the analysis included age (<35, 35–64, and >64 years), the respondent’s education level (at most secondary school vs at least high school), ethnicity (Kinh – the primary ethnicity vs other minor ethnicities), and partnership status (living with or without a partner). To examine their cigarette consumption behavior, we also included the number of years they smoked cigarettes (duration) and defined heavy smoking as >20 cigarettes per day.

### Statistical analysis

We employed a Latent Class Analysis (LCA) model to identify purchase behavior classes among cigarette smokers. We included the following dummy variables: quantity of cigarettes purchased (pack vs loose/carton), cigarette brand (domestic vs international), cigarette price tier (low vs medium/high), and place of purchase (kiosk vs other). Five different LCA models ranging from three-class to seven-class models were estimated. We fitted each LCA model with 1000 iterations using different random starting values to facilitate model identification. Also, we added the *CRITERION* and the *MAXITER* functions to reach convergence in all models. These LCA statistical analyses were conducted using PROC LCA in SAS 9.4 software.

For the selection of the best-fit model, statistical indices, including the likelihood ratio statistic G2, the Akaike information criterion (AIC), the Bayesian information criterion (BIC), and entropy (a measure of uncertainty), were compared across five models. Among these indices, lower G2, AIC, and BIC suggest a better balance between model fit and parsimony, whereas a higher entropy of at least 0.6 indicates a clearer class separation^11^. Based on the model fit criteria, interpretability, and parsimony of the models, we concluded that the four-class model of purchase behavior among cigarette smokers was the most appropriate (Supplementary file Table 1). After that, we assigned participants to classes based on their posterior probabilities (which refer to the probability of a participant being categorized in each class).

Further, we employed the principal component analysis (PCA), a mathematical linear transformation approach to reduce the dimensionality of high-dimensional data, aiming to preserve as much relevant information as feasible in a lower dimensional representation^11^. As such, we run the PCA to create a household wealth index based on housing characteristics and ownership of durable assets. We used the first component, which captured most of the variation of the variables included, to derive a component score, which was later used to categorize households into five quintiles. The first and the fifth quintiles included the poorest and the wealthiest households, respectively.

We employed a multinomial logistic regression model to examine the association between class memberships and covariates. All descriptive and regression analyses were weighted using individual weights. The weights were derived based on the sample selection probability (including non-responsive households and individuals) and adjusted for the post-sample stratification to ensure the sample was representative and minimize any possible bias^2^. The results are reported as an adjusted relative risk ratio (ARRR). These analyses were performed using Stata 17 software.

## RESULTS

Table 1 shows the tobacco smoking status across demographics, smoking, and purchase behavior characteristics. Compared with non-smokers, current smokers were more likely to be of older age groups, had lower education level, lived with a partner, and belonged to households with poorer socioeconomic positions. Among current smokers, nearly 80.0% smoked cigarettes, 30.3% smoked waterpipe, and 13.3% smoked more than one type of tobacco (data not shown). Among the cigarette smokers, over 70.0% bought cigarettes in a pack format, 63.5% smoked an international brand, and 68.0% bought cigarettes in the kiosk.

**Table 1:** Participant characteristics and their smoking and cigarette purchase behaviors*, GATS Vietnam 2015 cross-sectional study (N=3983)

*Characteristics*	*Current tobacco smoking status, n (%)*	
	No	Yes
	2080 (54.7)	1903 (45.3)
**Demographic**		
**Age,** mean (SE)	36.95 (18.05)	41.18 (14.04)
**Age** (years)		
15–34	786 (55.2)	479 (36.2)
35–64	977 (35.8)	1259 (57.9)
>64	317 (9.0)	165 (5.9)
**Education level**		
Secondary school or lower	1180 (58.9)	1367 (72.4)
High school or higher	897 (41.1)	535 (27.6)
**Occupation**		
White-collar	294 (12.1)	158 (7.1)
Blue-collar	1028 (53.0)	1355 (75.2)
Other	755 (34.9)	388 (17.7)
**Ethnicity**		
Major ethnicity	1870 (87.2)	1681 (88.0)
Other minor ethnicities	210 (12.8)	222 (12.0)
**Partnership status**		
Living with a partner	1474 (61.1)	1578 (79.0)
Living without a partner	605 (38.9)	325 (21.0)
**Household economic group**		
Tier I (poorest)	346 (18.9)	496 (26.3)
Tier II	350 (17.4)	418 (22.2)
Tier III	408 (19.3)	364 (20.2)
Tier IV	512 (23.8)	353 (18.2)
Tier V (wealthiest)	459 (20.6)	261 (13.1)
**Tobacco smoking behavior**		
**All participants** (N=3983)		
**Smoking status**		
Never smokers	1161 (36.6)	NA
Ex-smokers	919 (18.1)	NA
Current smokers	NA	1903 (45.3)
**Current smokers only** (N=1903)		
Cigarette smokers	NA	1518 (79.7)
Daily cigarette smokers	NA	1273 (83.5)
Occasionally cigarette smokers	NA	245 (16.5)
Waterpipe and shisha smokers	NA	560 (30.3)
Other tobacco smokers	NA	74 (3.4)
**Purchase behavior among cigarette smokers** (N=1518)		
**Amount of recent purchase**		
Loose cigarettes	NA	155 (10.8)
Cigarettes in pack	NA	1045 (71.3)
Cigarettes in carton	NA	247 (17.9)
**Cigarette brand by its origin**		
Domestic products	NA	535 (36.5)
International products	NA	912 (63.5)
**Cigarette brands by their price**		
Low (<10000 VND)	NA	560 (40.5)
Medium (10000–22500 VND)	NA	810 (54.6)
High (>25000 VND)	NA	74 (4.9)
**Location of purchased cigarettes**		
Kiosk	NA	1016 (68.0)
Street vendor	NA	395 (29.2)
Other	NA	40 (2.8)

* Sample size for individual characteristics may not be equal to the total due to missing values. Other tobacco products include hand-roll cigarettes, pipes, and cigars. SE: standard error. NA: not applicable. VND: 10000 Vietnamese Dongs about US$0.4 in 2015.

Among the four latent classes of cigarette purchase behaviors, the largest, referred to as ‘Class 1’, included smokers who were price-insensitive and purchased an international cigarette brand (44.4%) (Table 2). The other three latent classes were price-sensitive and included Class 2 (price-sensitive and purchased a domestic cigarette brand, 27.6%), Class 3 (price-sensitive and purchased cigarettes in street vendors, 18.6%), and Class 4 (price-sensitive and purchased either loose cigarette(s) or cigarette carton, 9.3%). The individuals within each latent class were homogeneous, and the model showed a good latent class separation with an entropy of 0.59 (Supplementary file Table 1). For example, the item-response probability of reporting being the Class 4 was 99% conditional on membership in the low-price tier latent class.

**Table 2:** The four latent classes of cigarette purchase behavior among participants of the GATS Vietnam 2015 cross-sectional study (N=1451)*

*Items*	*Latent class*			
	*Class 1*	*Class 2*	*Class 3*	*Class 4*
**Prevalence (%)**	44.4	27.6	18.6	9.4
**Item-response probabilities**				
Purchase by pack	0.69	0.98	0.71	0.00
Domestic brand	0.19	0.53	0.44	0.45
Low price tier	0.02	0.63	0.58	0.99
Purchase in kiosk	0.73	0.99	0.19	0.96

* Sample size may not be equal to the total of cigarette smokers (n=1518) due to missing values.

The distributions of demographic and tobacco smoking behavior variables for the different latent classes of cigarette purchase behaviors varied (Table 3). Overall, cigarette smokers in Class 1 were younger, whereas more of those in Class 4 had a lower education level. Compared to individuals in the other three latent classes, which were price-sensitive, we observed a higher percentage of smokers in Class 1 belonging to the two wealthiest groups. Besides, Class 4 smokers were older (mean age 43.95 years), had the longest smoking duration (25.3 years), and had the highest percentage of heavy smokers (57.2%).

**Table 3:** Demographic characteristics and tobacco smoking behavior by latent classes of cigarette purchase behavior among participants of the GATS Vietnam 2015 cross-sectional study (N=1451)*

*Characteristics*	*Latent class*			
	*Class 1 n (%)*	*Class 2 n (%)*	*Class 3 n (%)*	*Class 4 n (%)*
**Total**	652 (44.4)	424 (27.6)	244 (18.6)	131 (9.4)
**Age** (years), mean (SE)	38.45 (12.93)	41.61 (13.33)	39.80 (14.73)	43.95 (14.93)
**Age** (years)				
15–34	201 (43.3)	103 (33.9)	69 (41.4)	25 (31.2)
35–64	412 (53.2)	289 (61.4)	153 (52.4)	90 (61.1)
>64	39 (3.5)	32 (4.7)	22 (6.2)	16 (7.7)
**Education level**				
Secondary school or lower	430 (67.3)	293 (69.4)	181 (76.3)	108 (84.4)
High school or higher	222 (32.7)	131 (30.6)	63 (23.7)	23 (15.6)
**Ethnicity**				
Major ethnicity	634 (96.4)	378 (88.2)	215 (88.8)	117 (90.4)
Other minor ethnicities	18 (3.6)	46 (11.8)	29 (11.2)	14 (9.6)
**Partnership status**				
Living with a partner	499 (72.3)	359 (81.9)	195 (72.6)	118 (86.0)
Living without a partner	153 (27.7)	65 (18.1)	49 (27.4)	13 (14.0)
**Household economic group**				
Tier I (poorest)	105 (17.3)	110 (25.5)	85 (39.3)	35 (25.2)
Tier II	111 (17.3)	113 (26.8)	53 (20.9)	39 (31.8)
Tier III	123 (19.7)	82 (20.4)	41 (15.0)	24 (21.8)
Tier IV	163 (24.3)	73 (18.2)	39 (13.9)	23 (13.9)
Tier V (wealthiest)	146 (21.4)	43 (9.1)	26 (10.9)	10 (7.3)
**Number of smoking years,** mean (SE)	19.07 (12.57)	22.22 (13.38)	21.19 (14.38)	25.30 (14.87)
**Heavy smoking status**				
No	460 (74.4)	297 (73.0)	137 (53.5)	52 (42.8)
Yes	186 (25.6)	122 (27.0)	103 (46.5)	79 (57.2)

* Sample size may not be equal to the total of cigarette smokers (n=1518) due to missing values. SE: standard error.

Table 4 presents the multinomial logistic regression result to explore demographic and smoking behavior factors associated with classification relative to Class 1. The risk of belonging to Class 2 versus Class 1 was higher for respondents with higher education level (ARRR=1.75; 95% CI: 1.15–2.68) and belonging to minor ethnicities (ARRR=3.10; 95% CI: 1.27–7.52). Heavy smokers and those who had smoked for a longer period were more likely to belong to Class 3 and Class 4 versus Class 1. Those who belonged to poorer household economic groups had a greater relative risk of belonging to Class 2, Class 3, and Class 4 versus Class 1.

**Table 4:** Multinomial regression analysis examining purchase behavior classes among Vietnamese male cigarette smokers who participated in the GATS Vietnam 2015 cross-sectional study (N=1241)

*Variables*	Latent class*					
	*Class 2*		*Class 3*		*Class 4*	
	*ARRR*	*95% CI*	*ARRR*	*95% CI*	*ARRR*	*95 % CI*
**Age** (years)						
15–34 ®						
35–64	1.23	0.72–2.10	0.79	0.43–1.48	0.55	0.24–1.27
>64	1.11	0.36–3.36	0.74	0.26–2.17	0.34	0.07–1.67
**Education level**						
Secondary school or lower ®						
High school or higher	**1.75**	**1.15–2.68**	1.13	0.68–1.88	0.76	0.36–1.61
**Ethnicity**						
Major ethnicity ®						
Other minor ethnicities	**3.10**	**1.27–7.52**	2.28	0.80–10.00	2.82	0.74–10.85
**Partnership status**						
Living with a partner ®						
Living without a partner	0.63	0.37–1.05	1.24	0.71–2.17	0.69	0.27–1.77
**Household economic group**						
Tier I (poorest) ®						
Tier II	1.24	0.73–2.09	0.65	0.34–1.23	1.74	0.79–3.85
Tier III	0.77	0.47–1.26	**0.35**	**0.19–0.65**	0.86	0.37–1.98
Tier IV	**0.44**	**0.25–0.76**	**0.30**	**0.15–0.61**	0.55	0.24–1.25
Tier V (wealthiest)	**0.25**	**0.13–0.49**	**0.27**	**0.12–0.61**	0.36	0.12–1.03
Number of smoking years	1.01	0.99–1.03	**1.02**	**1.001–1.05**	**1.05**	**1.02–1.08**
**Heavy smoking status**						
No ®						
Yes	0.96	0.66–1.40	**2.33**	**1.51–3.58**	**2.94**	**1.71–5.06**

* Class 1: price-insensitive and international brand smokers as the reference category. ARRR: adjusted relative risk ratio. Bold values denote statistical significance at p<0.05. ® Reference categories.

## DISCUSSION

In this study, we identified four latent classes of Vietnamese male cigarette smokers with discernible patterns of purchase behavior: smokers who were price-insensitive and purchased an international cigarette brand; price-sensitive and purchased a domestic cigarette brand; price-sensitive and purchased cigarettes in street vendors; and price-sensitive and purchased either a loose cigarette or cigarette carton. The results yielded classes with high homogeneity and high-class separation. These allow us to disentangle heterogeneous groups of smokers based on their purchase behaviors using their sensitivity to prices, cigarette brand types and quantity of cigarettes purchased, and associated sociodemographic differences.

We found that 44% of the cigarette smokers belonging to Class 1 were of higher economic group and smoked international brands, which were priced at 1.5 times the cost of domestic brands (14.31 thousand VND compared to 9.67 thousand VND, data not shown). Our finding is consistent with an earlier qualitative study observing that Vietnamese smokers of higher socioeconomic status engage in the consumption of finer and more expensive tobacco, while smokers of lower socioeconomic status preferred cheaper tobacco brands^12^. Further, over a quarter of participants in an earlier study incorrectly believed that international brand cigarettes could be less harmful than domestic brands^13^. It could be explained by the common usage of misleading descriptors like ‘light’, ‘low-tar’, or ‘mild’ in these international brands, especially in Low- and Middle-Income countries like Vietnam, where the use of misleading words on cigarette packages is not regulated^6,14^. Also, earlier research indicated that cigarette smokers rated better taste, less harsh and dry if they smoked a pack displaying a premium brand^15^.

However, by using international brands with more expensive prices, the price-insensitive smokers need to pay more if tobacco taxation increases. The ad valorem system (VAT) of tobacco taxation in Vietnam means that with the same proportion of cigarette tax increase, the increase in cigarette prices of international brands will be larger than in domestic ones. In this case, the increase in taxation could help to reduce differences in smoking behaviors across different socioeconomic groups since the poorer smokers, who allocate a larger percentage of their income to cigarettes, would smoke less with increased cigarette prices than wealthier smokers, who smoke international brands with more increase in prices. Thus, a tobacco tax increase could not be a regressive policy, as usually mentioned by the tobacco industry^16,17^.

For the three price-sensitive classes (Class 2–4), their choice of cigarette brand is predicated on price, as was found in a previous study in Vietnam^18^. The negative association between each price-sensitive class and household income is unsurprising since more than half of price-sensitive cigarette smokers belong to the two poorest household economic groups. The large percentage of smokers who were classified as price-sensitive contributes to the evidence pointing out that a tobacco taxation increase in Vietnam might be effective in decreasing smoking prevalence. Price increases and higher taxation are cost-effective ways to both reduce cigarette consumption among current smokers and discourage smoking initiation among non-smokers^19-21^. A previous study confirmed that a 10% increase in cigarette price would decrease consumption by around 1% in Vietnam, reducing the number of male smokers by approximately 270 thousand^22^. With around 15 million smokers in Vietnam, such a decline would benefit society and individuals, especially the poorer group^22,23^.

Even though the increase in tobacco taxes could play an important role in reducing cigarette smoking demand, the current cigarette prices have become more affordable in Vietnam, standing as a massive barrier to tobacco control efforts. According to the WHO, the percentage of income required to purchase 100 packs of the-most-sold-brand of cigarettes in Vietnam had decreased from 9.3% to 4.3% of the gross domestic product (GDP) per capita between 2005 and 2016^24^. This greater affordability that has existed for nearly a decade has surely contributed to maintaining Vietnam’s status as one of the countries with the highest smoking prevalence among men globally^25^.

Furthermore, we found some behaviors of cigarette smokers when purchasing from street vendors (Class 3) or loose/carton smokers (Class 4). It could be partly explained by the cultural custom, as Vietnamese smokers who gather in the street vendor stores (because of their availability and easy accessibility) share cigarette sticks and smoke together^12^. They also report smoking more than usual in such gatherings compared to when they smoke alone because of the initiation of social interaction and peer pressure/impact^12,26,27^. Further, these street vendor places are yet to be covered by the smoke-free policies in Vietnam; thus, people could smoke freely here compared to other places where this policy took effect^8^. Therefore, to enhance the tobacco control policies impact, smoking as a social practice should be considered, potentially leading to an extension of smoke-free places, especially the places where smokers typically gather^28^. Further, the Vietnamese government should consider preventing price minimization strategies, as those behaviors of buying cigarettes in bulk or single cigarette sticks could happen more frequently, as observed in other countries, especially when tobacco taxation increased^29^.

### Strengths and limitations

This study utilized the Latent Class Analysis method, which allows us to identify homogenous unobserved subgroups of male smokers with different purchase behaviors in Vietnamese^30^. LCA has been widely employed in previous studies to identify subgroups of people with substance abuse, including tobacco^31-33^, drugs^34^, and alcohol^35^. If longitudinal panel data are available, this latent class method could be replicated to examine how purchase behavior changes over time (using latent transition analysis), potentially to be used to compare changes related to implementing a tobacco control policy. However, this study does have several limitations. The cross-sectional nature of this study might not reflect the dynamic cigarette purchase patterns over time or represent their regular purchase behaviors since smokers were asked about their most recent purchase only. Also, recall bias could exist when answering questions related to smoking behavior, number of cigarettes smoked, or smoking duration. The study only investigated the association between smokers’ purchase behavior and their demographic and cigarette smoking-related factors among Vietnamese males. Meanwhile, various dimensions could affect their purchase behavior, such as social or peer influences, cultural norms, or commercial impact, and how these interact, and we have not measured these dynamics. However, knowledge about price sensitivity is beneficial when it comes to tailoring interventions for different groups. Our findings also could not represent the whole population, particularly females, even though the number of Vietnamese female smokers was only about 1%.

## CONCLUSIONS

The distinct purchase patterns among male cigarette smokers in Vietnam, which are influenced by differing sensitivities to price and diverse economic backgrounds, emphasize the importance of adopting a comprehensive approach to tobacco control in Vietnam. This approach should encompass interventions aimed at targeting policy intervention related to escalation through taxation, altering individual smoking behaviors, and reshaping social norms related to cigarette purchasing and consumption.

## Supplementary Material



## Data Availability

The data supporting this research are available from the following source: https://www.cdc.gov/tobacco/global/gtss/index.htm
