# Supplementary material for: Patterns and determinants of tobacco purchase behaviors among male cigarette smokers in Vietnam: A latent class analysis
Source: Tob Induc Dis. 2024 Jun 4;22:10.18332/tid/187869. doi: 10.18332/tid/187869 (PMC11149401; doi:10.18332/tid/187869)
Supplement: Supplementary file 1 [file TID-22-98-s1.pdf]

Supplementary

Supplement 1: Comparison of LCA models with different latent classes based on model selection statistics. (a cross-sectional study, GATS Vietnam 2015, n=1451)

Number of latent classes	Maximum log-likelihood	df	G^2	AIC	BIC	ENTROPY
3	-29308301.60	1	33276.27	33304.27	33378.19	0.53
4	**-29291705.44**	**-4**	**83.95**	**121.95**	**222.27**	**0.59**
5	-29291663.49	-9	0.06	48.06	174.78	0.36
6	-29291663.49	-14	0.06	58.06	211.18	0.42
7	-29291663.49	-19	0.06	68.06	247.58	0.35

LCA = latent class analysis; AIC = Akaike information criterion; BIC = Bayesian information criterion

Supplement 2: Class membership probabilities for all LCA models. (a cross-sectional study, GATS Vietnam 2015, n=1451)

Number of latent classes	Class membership probabilities						
	1	2	3	4	5	6	7
3	0.64	0.17	0.19				
4	**0.40**	**0.08**	**0.26**	**0.26**			
5	0.30	0.18	0.22	0.11	0.19		
6	0.22	0.11	0.02	0.18	0.11	0.36	
7	0.25	0.03	0.12	0.13	0.19	0.19	0.09

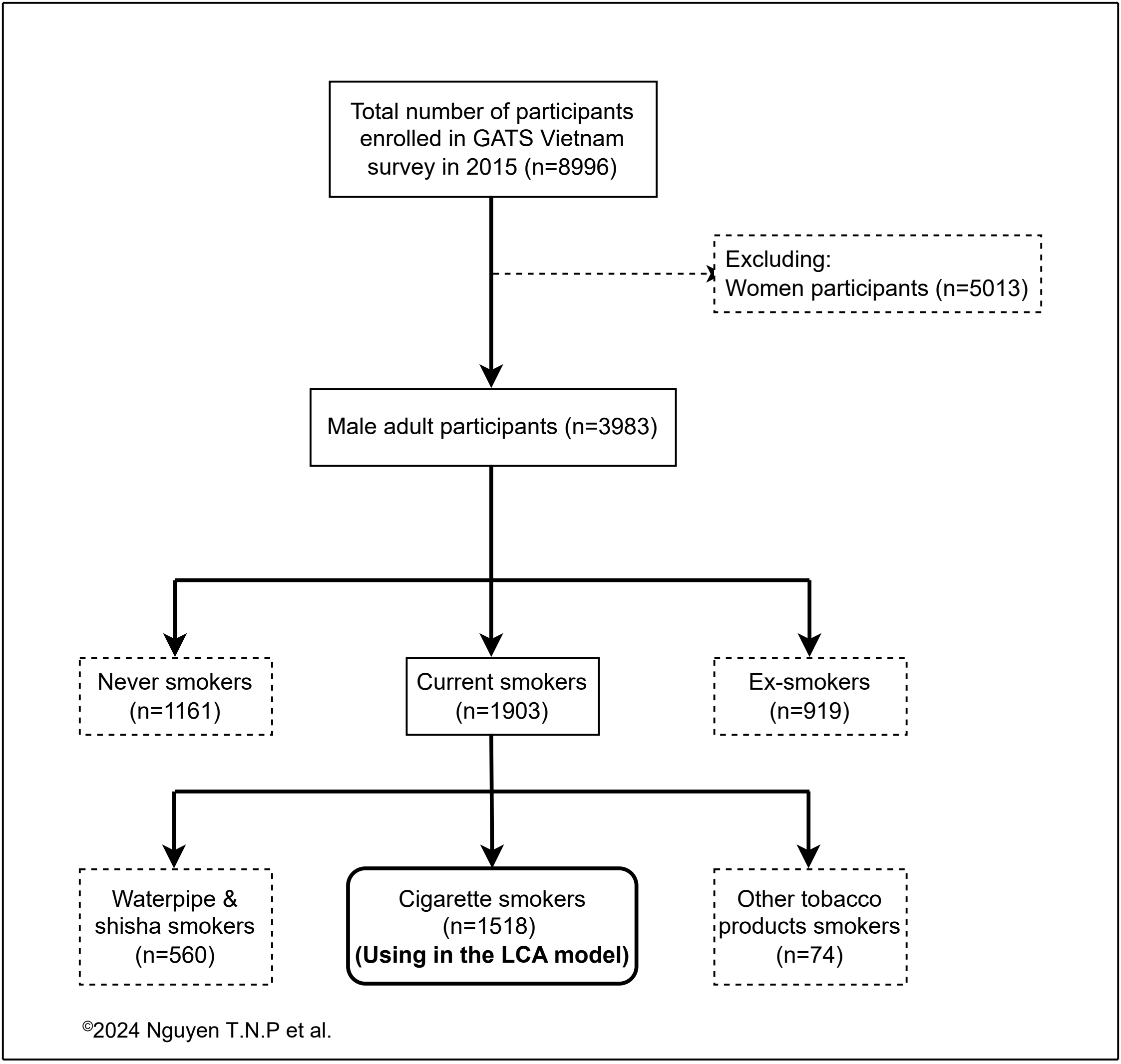
Total number of participants enrolled in GATS Vietnam survey in 2015 (n=8996)
Excluding:
Women participants (n=5013)
Male adult participants (n=3983)
Never smokers (n=1161)
Current smokers (n=1903)
Ex-smokers (n=919)
Waterpipe & shisha smokers (n=560)
Cigarette smokers (n=1518)
(Using in the LCA model)
Other tobacco products smokers (n=74)
©2024 Nguyen T.N.P et al.
